# Early Impact of SARS-CoV-2 Pandemic on Immunization Services in Nigeria

**DOI:** 10.3390/vaccines10071107

**Published:** 2022-07-11

**Authors:** Tene-Alima Essoh, Gbadebo Collins Adeyanju, Abdu A. Adamu, Alain Komi Ahawo, Desquith Aka, Haoua Tall, Aristide Aplogan, Charles S. Wiysonge

**Affiliations:** 1 Regional Directorate, Agence de Médecine Préventive (AMP) Afrique, Abidjan 08 BP 660, Côte d’Ivoire; htall@aamp.org (H.T.); aaplogan@aamp.org (A.A.); 2 Psychology and Infectious Disease Lab (PIDI), University of Erfurt, 99089 Erfurt, Germany; gbadebo.adeyanju@uni-erfurt.de; 3 Center for Empirical Research in Economics and Behavioral Science (CEREB), University of Erfurt, 99089 Erfurt, Germany; 4 Bernard Nocht Institute of Tropical Medicine (BNITM), 20359 Hamburg, Germany; 5 Cochrane South Africa, South African Medical Research Council, Cape Town 7500, South Africa; abdu.adamu@gmail.com (A.A.A.); charles.wiysonge@mrc.ac.za (C.S.W.); 6 Division of Epidemiology and Biostatistics, Department of Global Health, Faculty of Medicine and Health Sciences, Stellenbosch University, Cape Town 7505, South Africa; 7 Gavi, The Vaccine Alliance, Le Grand-Saconnex, 1218 Geneva, Switzerland; kahawo@gavi.org; 8 Directorate of Coordination of the Expanded Program on Immunization, Abidjan TF 599 BD, Côte d’Ivoire; aka.desquith2017@gmail.com; 9 HIV and Other Infectious Diseases Research Unit, South African Medical Research Council, Durban 4091, South Africa

**Keywords:** early impact, COVID 19, pandemic, SARS-CoV-2, immunization services, Nigeria, influence, access, uptake, vaccine confidence

## Abstract

Background: By 11 March 2022, there were 450,229,635 coronavirus disease (COVID-19) cases and 6,019,085 deaths globally, with Nigeria reporting 254,637 cases and 3142 deaths. One of the essential healthcare services that have been impacted by the pandemic is routine childhood immunization. According to the 2018 National Demographic and Health Survey, only 31% of children aged 12–23 months were fully vaccinated in Nigeria, and 19% of eligible children in the country had not received any vaccination. A further decline in coverage due to the pandemic can significantly increase the risk of vaccine-preventable-disease outbreaks among children in Nigeria. To mitigate such an occurrence, it is imperative to urgently identify how the pandemic and the response strategies have affected vaccination services, hence, the goal of the study. Methods: The research method was qualitative, including in-depth interviews of healthcare workers and focus group discussions (FGDs) with caregivers of children aged 0–23 months. We selected one state from each of the three zones of Nigeria: northern, central, and southern. Within each state, 10 local government areas and 20 healthcare facilities were purposively selected. In each facility, 10 healthcare workers were invited for interviews. Overall, 517 healthcare workers were interviewed. For the focus group discussion, 30 communities were selected. Within each selected community, six consenting caregivers were included. Overall, 180 caregivers participated. The data were analyzed using thematic inductive content analysis. Results: Three significant impacts that were observed are: difficulties in accessibility to immunization services, declining immunization demand and uptake among caregivers due to varying factors, and erosion of vaccine confidence among both caregivers and healthcare workers. Movement restriction and lockdown had numerous major impacts, such as decreased general healthcare service delivery, increased transportation costs, fewer engagements that promote vaccine uptake, and cessation of mobile vaccination campaigns that target hard-to-reach communities. Moreover, misinformation, conspiracy beliefs about the pandemic and COVID-19 vaccines, and risk perception negatively influenced general vaccine confidence. Conclusion: The results of this early impact study show that immunization was directly affected by the pandemic and provide insights into areas where interventions are needed for recovery.

## 1. Introduction

Coronavirus disease 2019 (COVID-19) is a viral disease that emerged in December 2019 [1]. It has an incubation period of 2–14 days, but can be transmitted by both symptomatic and asymptomatic carriers [2]. Its transmission is through droplets or contaminated surfaces. The basic reproductive number (R_o_) varies across settings, but current estimates suggest that it ranges from 2.24 to 3.58 [3]. The high transmissibility of this disease has contributed to its rapid global spread, with the disease now affecting nearly all countries worldwide [4]. According to the World Health Organization (WHO), by 11 March 2022, there were 450,229,635 cases including 6,019,085 deaths globally, with Nigeria reporting 254,637 cases and 3142 deaths [4].

COVID-19 has now spread to all 54 African countries, with the continent currently accounting for about 5% of all global cases [4]. A modeling study of the population in WHO Africa Member States predicted 22% of COVID-19-infected people to be from the region within the first year, with an estimated 37 million developing clinical symptoms and 150,078 deaths [5]. One of the consequences of such widespread community transmission of COVID-19 is its potential to overwhelm healthcare services, thus draining healthcare resources such as facility space, healthcare workers (HCWs) and funds [6]. This is because confirmed cases require isolation, and moderate and severely ill cases need hospitalization, and sometimes intensive care. It is estimated that 4.6 million COVID-19 cases will be hospitalized in Africa, out of which 139,521 will require oxygen and 89,043 will require intensive care support [5]. This will invariably necessitate a significant portion of the continent’s healthcare workforce to be diverted to COVID-19-related tasks. To minimize community transmission, African countries implemented several response strategies [7]. These strategies included lockdown at national and sub-national levels, in addition to behavior change interventions such as handwashing and the use of face coverings among others [8]. Lockdowns are restrictive because they prevent the movement of people and goods, and this can have an impact on health systems by reducing access to and uptake of healthcare services, and confidence in the healthcare system.

One of the essential healthcare services that have been impacted by COVID-19 is routine immunization [9], putting millions of children at risk of vaccine-preventable diseases (VPD) such as measles, polio, diphtheria, tetanus, and pertussis [9]. In African countries, the disruption of immunization services could have a much higher impact on child health than in most countries of the Northern Hemisphere, because immunization coverage on the continent was sub-optimal before the pandemic [10]. Coverage with the third dose of diphtheria-tetanus-pertussis containing vaccines (DTP3) in the WHO African Region has stagnated at 76% since 2016 [10], and about 26 countries (out of the 47 countries) in the region still have national DTP3 coverage of less than 90% [11].

Nigeria, with a DTP3 coverage of 57%, is among those projected to have the highest number of COVID-19 cases [11]. According to the 2018 National Demographic and Health Survey (NDHS), only 31% of children aged 12–23 months have been fully vaccinated in the country, with urban and rural coverage rates of 44% and 23%, respectively. Additionally, 19% of eligible children have not received any vaccination. A further decline in immunization coverage due to COVID-19 could significantly increase the risk of VPD outbreaks among children in the country.

Experience from previous disease outbreaks such as the 2014–2016 Ebola virus epidemic in West Africa, have shown that exacerbation of morbidity and mortality often occur because of the interruption of healthcare services [12]. In 2015, a study in Guinea reported multiple epidemics of measles following the destabilization of public healthcare services due to the Ebola outbreak [13].

In the WHO African Region, in addition to service disruption, demand for services was also affected, mainly due to rumors about COVID-19 vaccines [14]. In this region, vaccine confidence was identified as one of the top three pressing challenges during the pandemic [15]. The importance of vaccine confidence in global health is increasingly recognized [16]. WHO conceptualizes vaccine confidence within the broader framework of “vaccine hesitancy” [17]. Vaccine hesitancy, defined as a delay in the acceptance or refusal of vaccines despite the availability of immunization services, is a complex phenomenon, varying across time, place, and vaccines [17].

The Strategic Advisory Group of Experts on Immunization (SAGE) working group defined three factors, complacency, convenience and confidence (the 3C model), that influence vaccine hesitancy [17]. In this model, vaccine confidence reflects trust in vaccine safety and effectiveness; the immunization system (including health services and HCWs); and the motivation of policymakers who makes decisions on which vaccines are provided [17]. Crises such as the COVID-19 pandemic can influence public trust in immunization and thus affect confidence in vaccines [16]. A reduction in vaccine confidence can decrease vaccine uptake [16].

To avoid a surge in VPD and child mortality in Nigeria, it is urgent for policymakers to identify how the COVID-19 pandemic and response strategies are affecting childhood vaccination uptake, including access to and public confidence in vaccines. This will help to mitigate further disruption of immunization services in Nigeria. In addition to confidence and other behavioral insights, structural factors impacted by the lockdowns (a pandemic response strategy) could potentially become an even bigger contributor to increased incidences of VPD [7].

The relationship between COVID-19 and the immunization system is complex, and several mechanisms through which the pandemic can affect immunization services have been proposed [18]. However, there is a dearth of empirical evidence on the factors that affected recommended childhood vaccines during the COVID-19 pandemic in Nigeria. In addition, very few studies have used multiple qualitative data sources to explicitly describe system-wide mechanisms through which these factors are interrelated. Therefore, the goal of this study was to identify how the pandemic and response strategies have affected vaccination services in Nigeria.

## 2. Methodology

### 2.1. Study Design

**Figure 1:**
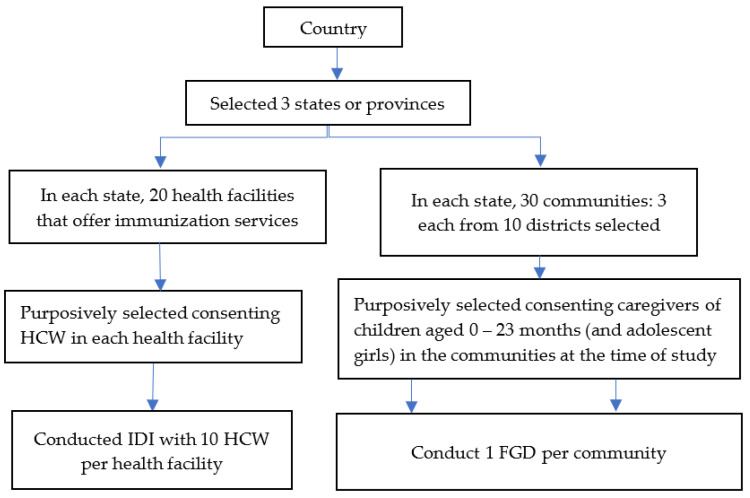
Study design flow.

The study used a mixed qualitative method including in-depth interviews (IDIs) and focus group discussions (FGDs). This design enabled the collection of different but complementary data types from two primary sources, i.e., caregivers and HCWs. The IDIs targeted HCWs, while FGDs focused on caregivers. Figure 1 describes the study design framework.

### 2.2. Study Setting

Nigeria was chosen because it is one of the countries in Africa most impacted by the pandemic, and it had also introduced measures in response to the pandemic. The study was conducted in Kaduna, Plateau, and Osun states. Within each state, 10 local government areas (LGAs) were purposively selected, and 2 healthcare facilities that offer immunization services in each of the LGAs were included in the IDIs. Six caregivers of children aged 0–23 months or adolescent girls in 10 communities in each of the states participated in FGDs.

### 2.3. Sampling and Sample Size

A simplified quota sampling approach was used to select caregivers and HCWs. The selected states were a mix of those with historically high and low immunization coverage levels based on predefined inclusion criteria and consideration of geographical representation, such as states that implemented a lockdown as part of their COVID-19 pandemic response strategy, facilities that offered immunization services for at least one year before the pandemic, and caregivers of children aged 0–23 months or adolescent girls. The states selected were: Kaduna (Northern Zone), Plateau (Central Zone) and Osun (Southern Zone).

The participants in the IDIs came from 20 health facilities that offer immunization services in 10 purposively selected LGAs per state. A maximum of 10 consenting HCWs were interviewed in each health facility, and in those who did not have 10 HCWs, all eligible HCWs were interviewed. Overall, a total of 517 HCWs were interviewed. At least 6 consenting caregivers of children aged 0–23 months or adolescent girls participated in the FGDs in 10 communities per state, resulting in a total of 180 participants from 30 communities. The samples were purposively guided to keep the focus on the characteristics of the populations that were of interest to the study.

### 2.4. Data Collection Process

Prior to data collection, study participants were provided with an information sheet on the study, stating the goals and expected outcomes, to enable risk analysis, if any. Informed consent was obtained from all study participants. The data collection was conducted between 3 August and 30 September 2021. Guides were used to lead the IDIs and FGDs. The IDIs, which lasted an average of 20 min, explored HCWs’ opinions regarding factors that affected caregivers’ demand for childhood vaccination services among caregivers during the COVID-19 pandemic. The IDIs and FGDs were audio-recorded after obtaining consent from the participants. Both the interviewer and interviewee maintained a distance of at least 1.5 m and wore face coverings, as required by the COVID-19 guidelines. Between six and nine participants were present at each FGD, which explored caregivers’ opinions on the impacts of the pandemic on vaccination services. All qualitative data were transcribed verbatim and stored in a password-protected drive.

### 2.5. Measures

The IDI and FGD guides explored topics such as: knowledge about COVID-19 (“Tell me what you know about COVID-19?”); knowledge of childhood diseases and immunization (“What childhood diseases are common in this community and what do you feel about immunizing your children?”); effect of the pandemic and the response strategies on access to childhood immunization (“How did the COVID-19 pandemic and response strategies affect people in your community and were you able to vaccinate your children during the pandemic? If not why?”); effects of the pandemic on the general healthcare system (“Tell me how the pandemic affected healthcare services in this community?”); sources of vaccination information (“How do you get information about vaccines and vaccination?”); and main reasons for low vaccination uptake during the pandemic (“what are the reasons that caregivers were able or not to receive immunization for children?”).

### 2.6. Data Analysis

The data were analyzed using thematic inductive content analysis. Main themes were identified after analyzing each individual transcript and then categorized based on the aggregation of subject outcomes. Transcribed data were coded as follows: the IDIs were coded as HCW001 and HCW002, while the FGDs were coded as FGD001 and FGD002. The analysis resulted in the development of themes and sub-themes based on the impacts of the pandemic on vaccination services.

## 3. Results

### 3.1. Impacts of COVID-19 Pandemic on Accessibility to Immunization

#### 3.1.1. Decrease in Healthcare Service Delivery

Healthcare service delivery, including immunization, was negatively affected by the pandemic from: both demand (caregivers) and supply (HCWs/facilities) perspectives. Fewer HCWs were able to go to work due to the pandemic (supply side), and caregivers were reluctant to go for immunization due to the pandemic (demand side). Some caregivers assumed that immunization services were not available in the facilities, and others were afraid of going to the facility because of infection risks. In addition, HCWs were reluctant to go to the facility because of the non-availability of consumables and vaccination materials:

*“The lockdown affected people and most caregivers were not able to visit the healthcare facilities, even here there are no vaccines and other drugs to administer, due to the COVID-19 pandemic”—HCW (Narayi PHC). “We are just trying to improvise now, at least to see how we can maintain the clients” HCW (Rayfield PHC). “People are scared of coming to the hospital because they read what is happening about the pandemic”—Caregiver (FGD: Yelwa, Iloko, Ijebu-Ijesha,Yekemi…communities).*

#### 3.1.2. General Fear of Overcrowding, Especially among Caregivers with Pre-Existing Conditions

It was difficult for caregivers who had pre-existing health conditions to go through all of the rigorous COVID-19 protocols at health facilities without inducing health crises. The rigor of the protocols at health facilities, such as wearing a face mask, social distancing, washing of hands, 1.5-m distancing, etc., created an overcrowded space and long waiting times, which induced fatigue in caregivers. They strictly avoided healthcare facilities, and, therefore, their children were not vaccinated. However, this behavior or fear of crowds was not limited to caregivers, since HCWs also expressed concerns:

*“It is difficult for clients who are suffering from other illnesses to come and follow all the sanitary protocols—HCW (Naray, Oja Timi, Oriade PHCs…). “Some healthcare facilities were not giving PPE to protect HCWs against COVID-19. so, we were exposing ourselves to so many dangers in crowded situations” HCW (Godogodo, Barkin Ladi, Foron, Kaduna south, Jema’a PHCs…). “There are not even enough face masks in the hospital, we are just managing, and I will come and expose myself because of what?” HCW (Agban, Igbona, Samaru…PHCs).*

#### 3.1.3. Lockdown and Movement Restrictions to Curb Community Transmission of COVID-19

One of the response strategies introduced by the Nigerian government was restricting the movement of persons, goods, and services. HCWs and caregivers, in particular, found it difficult to access healthcare facilities due to lockdown or movement restrictions. In some cases, there was a dusk-to-dawn curfew. The majority of IDI and FGD respondents cited the restriction of movement owing to lockdown as the major barrier to caregivers’ access to immunization services:

*“We are restricted in offering immunization services. So, it is one of the major reasons for decline in services”—HCW (Baban Dodo, Badiko, Kawo, Oba-Ife, Bokkos, Jos North…PHCs). “…it affected immunization services because we had total lockdown and transportation was not easy. So HCWs were found it difficult to come to work”—HCW (Kakuri, Jarmi, Dengi, Shika, Alaye…PHCs). “The caregivers were not able to reach healthcare facilities due to the lockdown”—Caregivers (FGD: Okefia, Kuda, Kagoma… communities). “Our children did not take immunization because we feared going out”—Caregivers (FGD, Oba-Ile, Oke-Oniti… communities). “Since there was a ban, no movement of vehicle or motorcycle due to the lockdown, this affected the ability of many caregivers to go to the healthcare facilities”—HCW (FGD: Ijebu-Ijesha, Kajola, Barnawa…PHCs and communities).*

#### 3.1.4. Decrease in Social Interactions and Public Gatherings

Due to a decrease in social interaction and gatherings, immunization campaigns and other supplementary activities that promote vaccination demand were halted. This created the impression that even healthcare facilities and services such as immunization were not exempted from the shutdown caused by the pandemic:

*“Unlike before where ceremonies are done with huge gatherings, now it is no longer the same”—Caregiver (FGD, Badiko community)*

#### 3.1.5. Cost of Transportation

The cost of transportation increased rapidly when the lockdowns and movement restrictions were imposed by the federal government of Nigeria. As a result, it was expensive and difficult to commute to work. The lockdown and subsequent absence of commercial transportation triggered hyperinflation. The majority of the FGD and IDI participants cited transportation-related issues as a major barrier to service provision at healthcare facilities. This was not limited to caregivers only, since there was an absence of vehicles to transport vaccines:

*“…It affected healthcare services because of transportation. Some patients wanted to come to the facility but because they are far away, it was difficult for them to come due to problems of transportation” - HCW (Pankshin, Chip, Buruku…PHCs). “The healthcare facility was opened but most parents were not able to come because of transportation issues, especially costs”—Caregivers (FGD: Op, Gyel, Rantya, Zarazong, Timbol, Madubi…communities).“I have a patient who told me that she could not afford the transport fare to take her child to the facility…the charges are unbearable”—HCW (Imo PHC). “There was no vehicle for them to transport the vaccine from cold store and distribute to facilities”—HCW (Sekona PHC).*

#### 3.1.6. Lack of Personal Protective Equipment (PPE) Stalls Vaccination Services Based on Perceived Risks

Caregivers could not access immunization services due to the non-use of use of PPE or face coverings such as masks by HCWs. Lack of PPE, which is necessary for the protection of HCWs was frequently described as a barrier against positive risk perception by caregivers and consequently affected service provision in the facilities.

*“We, the HCWs are not well equipped. There was no provision of PPE for us to protect ourselves”—HCW (Olusokan, Shendam, Rukuba, Eleyele… PHCs). “Caregivers are afraid of coming to the healthcare facilities. Before attending to the caregivers, we need to wear the required PPE, because they are scared” – HCW (Oriade, Tundun-wada PHCs). “Many caregivers came to the facilities without their face masks, so we tell them to return home to get them, but many never return” – HCW (Eleyele PHC); Caregivers (FGD: Okefia community).*

#### 3.1.7. High-Handedness of Law Enforcement Agents

Participants of the FGDs singled out the violent actions of law enforcement agents trying to enforce the lockdown order as a barrier to accessing immunization services:

*“Amotekun, police, soldiers, they stopped us from going or coming to hospital”—Caregivers (FGD: Iloko, Lokoro, Narayi…communities). “They can’t come out because the police arrest the people who don’t stay at home”—HCW (Sekona PHC).*

#### 3.1.8. Low Uptake in Hard-to-Reach Locations (Suspension of House-to-House Outreach)

Immunization services for house visits were halted. The services were completely cancelled in many areas, especially in hard-to-reach (H2R) areas that relied upon household-to-household outreach or mobile campaigns for immunization. Most of the participants agreed that pre-pandemic mobile campaigns, especially those targeting H2R communities, were efficient. However, since the pandemic and lockdown, it has been difficult for HCWs to reach these needy communities:

*“The reason is that people don’t like to come to the hospital. We had to go and educate people about the importance of vaccination. So, due to the Lockdown, going from house to house is no longer feasible”—HCW (Chikun PHC). “We are appealing to the Government to provide ways of receiving the vaccines easily, in such a way that HCWs don’t even have to travel” – Caregivers (FGD: Oba-oke, Aloma, Sakadadi, Ungwan Dosa…communities).*

### 3.2. Impact of COVID-19 Pandemic on Uptake of Immunization Services

#### 3.2.1. Fear of Contracting COVID-19 and Fear of Forceful Vaccination against COVID-19

A major sub-theme that emerged as a barrier to immunization demand was safety. Caregivers described fears ranging from the facilities being at high risk of COVID-19 transmission, fear that HCWs were super spreaders, and confidence in the safety of the COVID-19 vaccines themselves. Caregivers reported fear of contracting the virus and fear that they could be forcefully vaccinated against COVID-19; hence, caregivers stayed away or avoided healthcare facilities altogether:

*“We are afraid of contracting the disease from the HCWs. We will go to get immunization for our children after the lockdown is relax and COVID-19 is gone”—Caregivers (IROJO community). “People were not able to come down because of fear. Since it is a hospital, caregivers argue that other patients visiting might carry infection…they say ‘eh no dey show for face’. They don’t know much about the virus and that is one of the fears they have that prevents them coming to the facility“—HCW (Godogodo, Saminaka…PHCs). “Some were thinking that we have added COVID-19 vaccine into the immunization services, so they refused to come” – HCW (Ologunna PHC).*

#### 3.2.2. Knowledge Gap

Some of the participants described COVID-19 as a nickname of the country where the first case was reported and assigned its symptoms to malaria, cold, and cough. This limited knowledge may not only be for COVID-19 but could be for vaccine-preventable-diseases, in general. Not only does limited knowledge affect risk perceptions, but it can also encourage skepticism about vaccine preventable diseases:

*“I don’t really know much about COVID-19 but what I heard is that it is like someone who feels malaria symptoms in their body, like weakness, cold, too much coughing and he can’t control himself…”—Caregivers (FGD: Ologunna community)*

*“It is a virus that does not belong here but where it was first reported in Wuhan (China)” – HCW (Eleyele PHC; FGD: Iloko community).*

#### 3.2.3. Misinformation and Disinformation about COVID-19 Pandemic and COVID-19 Vaccines

Due to disinformation and misinformation about the COVID-19 pandemic and COVID-19 vaccines, especially through social media, caregivers were afraid that they might be wrongfully considered positive for COVID-19, when they visited a health facility for childhood immunization. The fear stemmed from rumors that people were being taken to isolation centers for quarantine if they showed interest in vaccination activities or even went near healthcare facilities.

*“Most of our patients thought that when they come to the healthcare facility, we might create another scene by saying we would move them to the isolation center, so most of the caregivers who were supposed to bring their children for immunization were scared” – HCW (Oja-timi PHC). “Some of us think that if we go to the facility, our children might be vaccinated with COVID-19 vaccine”—Caregivers (FGD: Imo, Isale-oyo, Adejuwan, Jos south…communities).*

#### 3.2.4. Socio-Economic Impact of the Pandemic

The financial difficulties created by the inflation caused by the COVID-19 pandemic, either directly through the economic effect on cost of living or indirectly due to the resultant increase in the prices of services such as transportation, were described by participants as a barrier to caregivers’ demand for immunization services. The pandemic impacted both global and national economies, thereby affecting costs of everyday goods and services, including transportation, which is vital for access to vaccination facilities:

*“They don’t have money…some have no money for transportation” – HCW (Ologunna PHC). “It really affected them because some people could not find a motorcycle to transport them to the clinic, and even if they found one, they can’t afford it, because it is double the price before the pandemic and lockdown started”—HCW (Olodan PHC; FGD: Giwa, Kakuri, Gora, Gidan Galadima…communities). “All the COVID-19 sanitary protocols put in place made me stay home because it would have cost me a lot of money”—Caregivers (FGD: Ologunna community). “People didn’t want to go because they were short of food stuff……. we that don’t have money to buy food, how can we go for vaccination?”—Caregivers (FGD: Olodan community).*

#### 3.2.5. Lack of Solar Energy to Power Vaccine Storage Facilities

Vaccines are stored centrally, and they need to be distributed to healthcare facilities, particularly in rural areas, where the majority of healthcare facilities are situated. Due to the pandemic, the central storage facilities and the services were shut down, although, in some areas, the services were very sporadic. Similarly, refrigerators in healthcare facilities where vaccines are temporarily stored prior to being administered were without power, and with no alternative source of power, this meant that the cold chain of vaccines was not respected, and therefore, immunization services were disrupted: this hampered the preservation of vaccines and disrupted services. Therefore, a lack of alternative sources of power such as solar disrupted immunization uptake.

*“Electricity contributed to low uptake, because during the lockdown, some healthcare facilities didn’t have the electric power needed for vaccines storage. I think this is the things that really affected immunization services”—HCW (Ologunna, Iwaraja, Buruku…PHCs). “We don’t have anywhere to store vaccine during power cut, such as solar powered refrigerators, so caregivers didn’t bring their children for immunization”—HCW (Ologunna PHC). “Some facilities in rural areas where there are no solar refrigerators to store the vaccines were unable to provide immunization services”—HCW (Iso ege, Isale oyo PHCs).*

#### 3.2.6. Long Queues Due to Strict COVID-19 Sanitary Protocols

Due to strict adherence to COVID-19 sanitary protocols at healthcare facilities, such as social distancing and the use of nose and mouth coverings/masks, the numbers allowed in the vaccination area were reduced, thereby leading to longer waiting times, and this hampered the provision of services in the facilities:

*“We were not allowed to sit together as we used to before the pandemic started, and we were spending longer than before”—Caregivers (FGD: Imo community). “All of the HCWs were saying if you don’t use face masks, you can’t enter, even when we don’t have any, we had to go away to get a mask before they would see us” – Caregivers (FGD: Okefia, Ijebu Ijesha…communities).*

### 3.3. Impact of COVID-19 on Confidence in Immunization Services

#### 3.3.1. Lack of Confidence in COVID-19 Information and Poor Attitude among HCWs

HCWs did not trust the information that they received on COVID-19. The absence of proper training of HCWs on the COVID-19 pandemic was obvious. In addition, the poor attitude of HCWs affected immunization services. They were not motivated, and their hazard and pandemic-related allowances were not paid, which resulted in low morale and a poor attitude to work:

*“Not all HCW trusted the information provided that said once you practice the precautionary measures like social distancing, use of PPE and practicing personal hygiene like handwashing, you would be unlikely to be infected and when the COVID-19 vaccine also comes, you can take the vaccine and the chances of being infected are slim…if you are infected, the chances of severity are slim” – HCW (Oja timi, Ologunna PHCs). “They did not pay our allowances, so we are not motivated”—HCW (Kawo, Badarawa, Alomi…PHCs).*

#### 3.3.2. Risk Perception of COVID-19 Pandemic and Susceptibility to Infection at Healthcare Facilities

The caregivers’ risk perception limited attendance at immunization services because they were afraid of being infected with COVID-19. In previous outbreaks, fear of being infected was reported as the prominent suicide stressor. Therefore, the severity of the disease has become a concern, because it worsens emotional, cognitive, and behavioral responses:

*“People were even scared of going to the hospital because of reading what was happening and fear that hospitals were super spreader centers”—Caregiver (FGD: Yelwa, Sabon Tasha, Kaura, Olodon, Sekona…communities)*

## 4. Discussion

**Figure 2:**
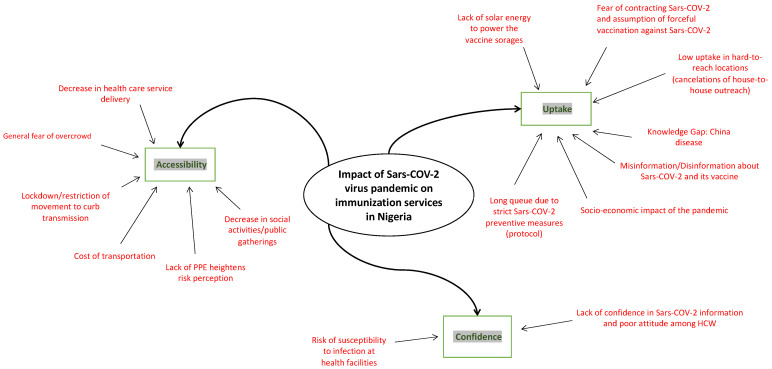
Components of the impact of the COVID-19 pandemic in Nigeria.

This study assessed immunization service uptake and the effects of policy strategies and measures taken to prevent community transmission in the context of the COVID-19 pandemic in Nigeria. Three significant factors were affected, i.e., accessibility to immunization service, a significant reduction in immunization demand and uptake among caregivers, and a lack of trust by caregivers and HCWs (Figure 2).

The results showed that accessibility to immunization services was a big barrier during the pandemic for the majority of participants, as were lockdown and restriction of movement, curfews, inflation, increased cost of transportation, economic recession due to the pandemic, suspension of mobile immunization campaigns to H2R communities, and lack of PPE for HCWs. Some of these factors were to be expected in a pandemic situation. However, the lack of provision of PPE, the lack of knowledge and training for HCWs, and the lack of alternative outreach measures for H2R communities created an impression of the absence of pandemic preparedness. Further, those involved in the healthcare system decision-making process in Nigeria were unprepared. In addition, they were not consulted on how the lockdown measure would impact healthcare services, including immunization. This may partially explain why many healthcare facilities were closed during the lockdown and demonstrates the vacuum or disconnect between public health decision-making structures (health professionals in the Ministry of Health and stakeholders or partners) and policymakers or politicians in Nigeria. Public health policy must rely on the advice of health professionals and behavioral experts, especially during pandemics and health emergencies, but the evidence suggests that this was not the case in Nigeria.

Secondly, demand for and uptake of immunization declined due to the pandemic, and countermeasure-related-factors, such as fear of contracting the virus and fear of the forceful vaccination of caregivers against COVID-19, fake news, misinformation and disinformation, poor knowledge, the lack of appropriate storage infrastructure (such as solar-powered refrigerators in facilities), the high-handedness of law enforcement agents, long waiting hours by caregivers, and the increased indirect personal costs of accessing care, such as the need to buy PPE and sanitizers and transport for each visit, contributed to the disruption of routine immunization uptake. These are largely consistent with some of the main reasons proposed by experts, to date, for the disruption of immunization services, primarily highlighting a combination of vaccine demand and supply factors [19].

The knowledge gap during the pandemic in Nigeria not only impacted the risk perception of the disease but also compounded existing vaccine hesitancy in general. Associating malaria with COVID-19 or thinking that the two diseases were the same by both caregivers and HCWs, as seen in this study, highlights a significant knowledge gap in Nigeria. This accentuates a previous study across Nigeria that showed that caregivers misunderstood VPD to include malaria [20]. In a state in the North-eastern region (Katsina) of Nigeria, some policymakers and healthcare system managers thought that oral polio vaccination provided immunity against all childhood diseases [20]. Therefore, the knowledge gap that created the belief that immunization can prevent all childhood diseases, including malaria or COVID-19, might lead to a lack of confidence in the efficacy of vaccines to prevent VPD and in the healthcare system. The knowledge gap found in this study is similar to that found in Sub-Saharan Africa (SSA), where the population has misconceptions about vaccines [21,22,23,24]. More work is still needed in SSA to improve the knowledge of VPD and determine the best communication method for vaccination information.

The lockdown exposed and worsened existing immunization inequities and uptake barriers. However, the risk of visiting healthcare facilities for immunization still outweighs the consequences of COVID-19 infection. A recent benefit–risk analysis modeling the impact of missed childhood vaccinations in Africa found that the benefit of averting vaccine-preventable infections far outweighed any possible COVID-19 morbidity and mortality associated with immunization clinic visits [10].

The confidence of HCWs and caregivers in the healthcare system was tested during the pandemic, and both contributed to the negative impacts on immunization services. HCWs’ attitudes were poor, and they were not motivated due to the lack of payment of their hazard and pandemic-related allowances, “They did not pay our allowances, so we are not motivated” – HCW (Ologunna, Kaduna North…PHCs). Further, the general mistrust of COVID-19 information shows a lack of confidence in the healthcare system or in training, as well as a significant lack of knowledge of diseases. “Not all HCW trust the information…about the Coronavirus and COVID-19 vaccine” – HCW (Oja timi, Ologunna PHCs). In addition, caregivers’ perception of the risk of infection was a major drawback to immunization uptake. Perceived inadequate infection prevention and control measures in healthcare facilities, including the lack of adequate PPE and other consumables, and measures, such as HCW training, may have created hesitation among caregivers and made the general population reluctant to access healthcare services, including immunization services. Interventions targeting confidence and safety in the healthcare system will be key for the recovery of uptake to the level in the pre-pandemic situation.

The results from this study demonstrate the gap in immunization messaging, highlighting the need for improved efforts at behavior change communication policies that convey key messages targeting caregivers to emphasize the importance of attending routine vaccination services, despite the COVID-19 pandemic and the response strategies for infection prevention [25,26]. Effective communication strategies will serve to counter misinformation, which has, unfortunately, been the bane of the pandemic and which may not disappear post-pandemic [27,28]. In addition, increased efforts are needed to optimize logistics at both local and national levels. This primarily includes supporting vaccine supply chains, reinforcing transportation or infrastructural networks (for both vaccine delivery and user access/travel), and providing access to hand-washing facilities and free face masks at healthcare facilities. Simple solutions such as aligning vaccination with other child healthcare services, as well as robust infection control procedures, will enable the safe provision of immunization services during the pandemic.

Alternative immunization delivery mechanisms such as using private pharmacies (as vendors) may be worth consideration. This will facilitate vaccine delivery closer to caregivers’ homes, thereby overcoming challenges of distance and transportation [29].

The non-probabilistic nature and representativeness of the study sample was a limitation observed, even though the included HCWs and caregivers were quasi-randomly selected from healthcare facilities. However, a sense of representation of the regions was achieved by equally distributing the study centers in the north, central, and south regions. In addition, the sample was made large to accommodate for saturation for a large population such as Nigeria.

As the COVID-19 pandemic persists, further studies are needed to understand in-depth causality between the pandemic, measures to counter the pandemic, and low immunization coverage in order to inform comprehensive vaccination intervention programs. Additionally, further research would be helpful to understand how these study results, in terms of accessibility, uptake, and confidence in immunization services, may have affected attitudes toward COVID-19 vaccine acceptance in Nigeria.

## 5. Conclusions

The goal of this study was to generate systematic context-specific evidence on the impacts of the COVID-19 pandemic on immunization services and uptake. A reduction in the accessibility of immunization services, low demand for immunization, and lack of confidence in the immunization system are the main discoveries of this study. The results explain not only how the COVID-19 pandemic negatively affected immunization services but also how it brought the health system to a near collapse. The national lockdown was the factor that had the most pronounced negative impact on immunization services in Nigeria during the COVID-19 pandemic. The restriction of movement led to a general decrease in healthcare service delivery, including immunization services. The restriction in movement also led to an increase in the cost of transportation and a decrease in immunization outreach activities. All of these amplified the already fragile vaccine confidence levels in some quarters. However, we also found that there are positive spinoffs from the pandemic. These include good sanitary practices such as frequent hand washing and cough hygiene.

This study contributes new knowledge on the early impacts of COVID-19 on immunization services and expands the current understanding of the extent of its influence by elucidating individual, social, and context-related factors affecting access, confidence, and uptake. The evidence generated can be useful for in-country immunization stakeholders and partners to inform the formulation and design of new strategies to minimize immunization system disruptions during COVID-19.

## Data Availability

The datasets used and/or analyzed during the current study are available from the corresponding author on reasonable request.

## Abbreviations

AMP	Agence de Médecine Préventive
DTP3	Third dose of diphtheria-tetanus-pertussis containing vaccine,
EPI	Expanded Program on Immunization
FGD	Focus group discussion
GAVI	Global Alliance for Vaccines and Immunization
H2R	Hard-to-reach
HCW	Healthcare workers
HPV	Human papillomavirus
IDI	In-depth interview
MoH	Ministry of Health
PHC	Primary health center
PPE	Personal protective equipment
Q&A	Question and answer
RI	Routine childhood immunization
SSA	Sub-Saharan Africa
UNICEF	United Nations International Children’s Emergency Fund
VPD	Vaccine-preventable diseases
WHO	World Health Organization
WUENIC	WHO/UNICEF Estimates of National Immunization Coverage
